# Lower peak knee joint kinetics during walking in patients with knee reconstruction for bone sarcoma compared to healthy controls

**DOI:** 10.7717/peerj.21201

**Published:** 2026-05-06

**Authors:** Hannah Margaret Rice, Merethe Lia Johansen, Patrick Mai, Anders Hjort Matthiasen, Joachim Thorkildsen, Ola Eriksrud, Tormod Skogstad Nilsen

**Affiliations:** 1 Department of Physical Performance, Norwegian School of Sport Sciences, Oslo, Norway; 2 Division of Cancer Medicine, Department of Clinical Service, Section for Cancer Rehabilitation, Oslo University Hospital, Oslo, Norway; 3 Department of Medical and Health Services, Vestre Viken Hospital Trust, Oslo, Norway; 4 Department of Sport Science and Physical Education, University of Agder, Kristiansand, Agder, Norway

**Keywords:** Gait analysis, Biomechanics, Joint kinetics, Rehabilitation, Osteosarcoma, Knee reconstruction

## Abstract

**Background:**

Osteosarcoma patients who have undergone prosthetic lower limb reconstruction following tumour resection walk at a slower preferred speed than healthy controls. In addition, they demonstrate lower peak isokinetic knee extension torques, which may explain the observed differences in sagittal plane knee joint kinematics during walking. However, mechanisms for this and the associated knee joint kinetics are not well understood, particularly at matched walking speeds.

**Methods:**

This observational case-control study compared sagittal plane lower limb walking gait characteristics between patients who have undergone prosthetic reconstruction and matched healthy controls across their preferred and matched walking speeds. Data were collected from 18 control participants and 17 patients while walking on a force-instrumented treadmill at the different speeds. Spatiotemporal variables, peak knee flexion angle, peak knee extensor moments (torques), and peak knee joint power were compared between groups.

**Results:**

Patients walked with a slower preferred speed than the control group. When comparing lower limb gait mechanics at matched speeds, patients demonstrated lower magnitude peak knee extensor moments than the control group, and these differences were greater at faster speeds. At the fastest walking speed, patients also displayed lower peak knee joint power compared to the control group. The differences in knee joint kinetics observed between groups may be due to an inability amongst patients to generate the magnitudes of knee extensor force that the control group can generate.

## Introduction

Osteosarcoma is a primary malignancy of bone, most prevalent amongst adolescents, with a 5-year survival rate of 60–75% reported in 2020 (Childs et al., 2024). Limb-saving surgery is performed for 80–95% of patients (Grinberg et al., 2020) and involves surgical removal of the bone affected by tumour with appropriate surgical safety margin followed by reconstruction, often with a segmental metal prosthesis (Grinberg et al., 2020). Osteosarcoma commonly develops on the metaphysis close to the knee joint (Takeuchi et al., 2019; Marulanda et al., 2008), such that it is often the knee joint that is replaced with a prosthesis. The highly invasive nature of the resection is inevitably associated with functional impairments. Individuals who have been treated for lower limb osteosarcoma show reduced motor performance (Kesting et al., 2015) and reduced ability in their daily activities, including walking (Sugiura et al., 2001). An impaired gait after resection may result in reduced physical activity and sports participation compared to healthy controls (Bekkering et al., 2010), leading to poorer health outcomes (Lee & Buchner, 2008).

There is limited understanding of how limb resection influences the mechanics of walking. A systematic review and meta-analysis found that individuals who have undergone prosthetic reconstruction following tumour resection walk at a slower speed, have a shorter stride length and a lower stride frequency than controls (Filis et al., 2022). The difference in speeds is reportedly 10–40% slower in patients than controls (Kim, Ryu & Jung, 2021; De Visser et al., 2000). A faster walking speed can be achieved by increasing stride length, stride frequency, or both, and this relies on increased sagittal plane knee joint kinetics as well as increased knee flexion (Chen, Kuo & Andriacchi, 1997; Holden, Chou & Stanhope, 1997). It has been reported that individuals who have undergone prosthetic reconstruction after tumour resection (PRaTR) (Carty et al., 2009), or total knee replacement, (McClelland et al., 2011) demonstrate less knee flexion during walking stance than controls, but these results are likely influenced by a slower self-selected walking speed amongst the patients.

In addition to altered knee kinematics (*i.e.,* movement) during walking, knee kinetics (*i.e.,* joint moments or torques, and powers) may also be impaired in PRaTR individuals, but this remains unclear as very limited information is available in PRaTR individuals regarding knee kinetics during walking. Lower magnitudes of knee joint kinetic variables have been observed in patients with knee osteoarthritis compared to a healthy control group (Zeni & Higginson, 2009). However, the osteoarthritis patient group self-selected a slower walking speed than the controls, thus, it is unclear whether the lower knee joint kinetics were a function of walking speed or the disease directly. Assessing walking gait at both preferred and matched walking speeds is required in order to improve understanding of the mechanisms of gait impairment.

Given that the mechanical demands of walking increase with increasing speed (Chen, Kuo & Andriacchi, 1997; Holden, Chou & Stanhope, 1997), any knee joint impairments may be exacerbated at faster speeds, resulting in differences in knee joint kinetics. This was evident amongst PRaTR patients who did not increase knee extension moments (torques) on the affected side when increasing walking speed from a preferred speed to a faster speed, yet did so on the unaffected side (Okita et al., 2014). This is indicative of some form of compensatory mechanism whereby more absolute mechanical work is conducted by the unaffected than affected side to achieve faster walking speeds. This previous study (Okita et al., 2014), however, did not have a control group, and the mechanisms and strategies used to achieve faster walking speeds could therefore not be directly compared with individuals without knee replacement. We have previously shown that tumour resection patients generate isokinetic knee extension torques that are approximately one-fifth of the magnitude observed in their control counterparts (Johansen et al., 2023), indicating severely impaired muscle function. This suggests that the previously reported compensatory mechanisms observed when walking at a faster speed may be due to an inability to generate the necessary knee extensor torques to achieve equivalent knee joint kinetics after resection, but this has not been studied directly. Comparison of knee joint kinematics and kinetics during walking between PRaTR individuals and controls at matched walking speeds is warranted, as this information can inform rehabilitation.

The aim of this study was to compare spatiotemporal gait characteristics and sagittal plane knee joint kinematics and kinetics at different walking speeds between individuals who have undergone prosthetic reconstruction after tumour resection (Resection Group) and healthy controls (Control Group). Sagittal plane knee joint mechanics were the main focus of the present study as previous results have shown that the Resection Group achieved considerably lower isokinetic knee extension torques (*i.e.,* sagittal plane) than the Control Group (Johansen et al., 2023), which would likely influence sagittal plane knee mechanics during walking. In addition, ground reaction force variables and sagittal plane ankle and hip kinematics and kinetics were compared to facilitate understanding. We hypothesized a slower preferred walking speed in the Resection Group and differences in sagittal plane knee kinematic and kinetic variables at this speed. When walking at matched speeds, we hypothesized that there would be no difference in spatiotemporal variables between groups, but that the Resection Group would demonstrate lower peak knee flexion angle, lower peak internal knee extensor moments, and lower peak negative knee joint power on the affected side than in the matched side of the Control Group. We further hypothesised that these differences would be exacerbated at faster speeds. In addition, we explored associations between knee extensor moments during walking and dynamometer-obtained knee extension torque to provide clinical insight into potential mechanisms.

## Materials & Methods

This observational case-control study was part of a larger study (Johansen et al., 2023) where eligibility criteria, surgical procedures, assessments of patient-reported outcome measures, and muscle strength assessments have been described in detail. Briefly, participants in the Resection Group were a minimum of 12 years of age; had undergone bone sarcoma surgery with insertion of a megaprosthesis in the distal femur or proximal tibia; a minimum of 12 months since surgery at the time of inclusion; reported no use of walking aids; had not experienced metastatic disease or local relapse and were not suffering from severe anxiety or depression. Participants in the Control Group were matched for year of birth and sex to participants in the Resection Group, had no history of any cancer, and had no walking limitations.

Participants in the Resection Group had undergone intra-articular resections, which were necessarily individualised based on tumour size and location. Information about which muscles were removed/released according to tumour location and further information about procedures post-surgery have previously been reported (Johansen et al., 2023). The study was approved by the Regional Committee for Medical and Health Research Ethics (reference number: 2017/1058). Written informed consent was provided by participants or their guardians provided prior to assessment.

### Participant characteristics

Demographic and treatment variables, such as age, time since surgery, chemotherapy status, tumour location, and prosthesis length, were obtained from hospital medical records. The lower extremity version of the Toronto Extremity Salvage Score (TESS) was used to assess self-reported physical disability. The questionnaire is based on 30 questions, yielding a total score of 0-100, where higher scores indicate greater autonomy.

### Gait assessment

In the present study, we compared walking gait between groups at each individual’s preferred speed and at “matched” walking speeds. It has previously been observed that patients walk more slowly than healthy controls by approximately 10–40% (Kim, Ryu & Jung, 2021; De Visser et al., 2000), where the difference is influenced by the tumour location (Kim, Ryu & Jung, 2021). In the present study, walking gait was assessed during treadmill walking at each participant’s preferred speed and at a speed that was 20% faster and 20% slower than preferred. This approach ensured that walking gait was assessed at an effort that was approximately equivalent for all participants, which was important in this study where a high degree of heterogeneity was expected within each group. This allowed comparison of gait mechanics at the preferred speed–relevant for daily living–and provided insight into the mechanisms that cause gait differences; *i.e.,* by assessing gait at matched speeds. An alternative approach would have been to select a fixed speed for all participants which would have helped to provide understanding of mechanistic differences but could have resulted in large variability in terms of how demanding and realistic the walking speed was for participants in the Resection Group. For the analysis at matched speeds, walking mechanics from the Resection Group’s preferred and faster speeds were compared with the Control Group’s slow and preferred speeds, respectively. In order to match the mean speeds per group, the fastest participants from the Control Group would be excluded if necessary, such that the Resection Group’s average preferred and fast speeds matched the Control Group’s slow and preferred speeds, respectively.

Seventy-eight reflective anatomical and tracking markers were secured on each participant to allow reconstruction of rigid segments representing the right and left foot, right and left shank, right and left thigh, and the pelvis, torso and upper extremities, using the marker set described in detail by Willwacher et al. (2016). A static trial was conducted with the participant’s feet facing in the direction aligning approximately with the positive anterior-posterior direction of the laboratory coordinate system. Walking was conducted at the preferred speed first in each case, followed by the slower speed and then the faster speed. Preferred speed was determined *via* the following protocol: participants were asked to walk 10 metres overground at the speed that felt most comfortable for them. They were not given instructions regarding how long they would need to sustain this speed. Following this, participants walked for five minutes on the Motek force-instrumented treadmill (M-Gait, Motion Force Link, Amsterdam, The Netherlands) starting at a speed slightly slower than their preferred overground speed. This speed was thereafter adjusted during walking based on feedback from each participant regarding what felt like the most “natural” walking speed for them. Participants were given 30 s to become accustomed to each walking speed and then walked for three minutes at each speed, during which 30 s of data were recorded. The 30 s of data were obtained at a non-specified time point, avoiding the first and final 30 s of the three-minute walking period. Participants were provided with a rest period of two minutes between each speed condition.

Assessment of knee extension strength and the results from this have been reported previously (Johansen et al., 2023). Knee extension strength was assessed using a HUMAC NORM (CSMi, Stoughton, MA, USA) isokinetic dynamometer. Assessments were conducted in a seated position with the dynamometer position adjusted according to each individual. The dynamometer was aligned such that the external rotation axis appeared visually to align with the knee joint flexion-extension axis. Participants were restrained using the dynamometer’s adjustable four-point belt, with investigators specifically focusing on securing the pelvis to the seat and the upper body against the back rest, to the extent possible. It was ensured that the pelvis was against the back rest when securing participants. Straps were used to secure the thighs on both the tested and non-tested side to the seat and participants were instructed to hold the handles located at the side of the seat. Participants completed three warm-up sets of three repetitions with increasing effort. Maximal knee extension torque was measured at three angular velocities (60° sec^−1^, 120° sec^−1^, and 180° sec^−1^) and was assessed within the participant’s knee range of motion, up to a maximum range of 80°. The order of angular velocities was from slowest to fastest, thereby maximising the quality of the data at the slowest speeds, where it was known from pilot testing that the higher angular velocities could be challenging for some participants. During testing, participants were asked to engage with maximal extension force from the start and through the full range of motion. Verbal encouragement was provided by the investigator during all attempts. During data extraction, force-muscle length curves were visually inspected by two investigators to ensure anomalous data were not included.

### Data processing

Kinetic data were collected using a force-instrumented treadmill (M-Gait, Motion Force Link, Amsterdam, The Netherlands, 1200 Hz), and kinematic data were collected using a 15-camera Qualisys system (Qualisys Inc., Gothenburg, Sweden, 240 Hz). Coordinate and force data were filtered with a second-order low-pass Butterworth filter with a cutoff frequency of 15 Hz (Mai & Willwacher, 2019) in MATLAB (MathWorks, Natick, MA, USA). The filtered data were then analysed in OpenSim software (Delp et al., 2007), *via* a customised MATLAB program. The Rajagopal model (Rajagopal et al., 2016) was used to represent the trunk and lower limbs, without modification. This model was developed using data from healthy, young adults with a mean age of 25.5 years (Handsfield et al., 2014). The knee joint is represented as a single degree of freedom joint, permitting knee flexion and extension. The model was scaled to the size of each participant using OpenSim’s scaling tool. Analysis of ankle, knee, and hip sagittal plane kinematics and kinetics were conducted using OpenSim’s Inverse Kinematics and Inverse Dynamics tools, respectively, where Inverse Kinematics results are filtered prior to Inverse Dynamics analysis. Quantification of spatiotemporal variables (ground contact time, stride time, and stride length and frequency) were conducted in MATLAB from the filtered ground reaction force data.

Data from the patients’ affected side and the corresponding matched control’s equivalent side were analysed for the main analyses. Data from the contralateral limb were also obtained and reported to aid understanding, where relevant. Twelve strides of data were analysed for each side per person, at each walking speed. These were the first 12 strides within the 30 s of recorded data after removal of outliers. Outliers were defined as trials in which the peak value of an outcome variable was more than three scaled median absolute deviations from the median, using the ‘isoutlier’ function in MATLAB. Kinematics were presented across each stride cycle, whereas kinetic variables were presented across each stance phase, and in each case data were time-normalised to 101 data points. For analysis of discrete variables, the following values were extracted: peak knee flexion angle during stance; peak internal knee extensor moment; peak negative knee joint power; peak ankle dorsiflexion angle; peak internal plantarflexor moment; peak hip extension moment; the first peak in vertical ground reaction force; peak braking force; peak propulsive force. These discrete variables were selected as they occurred during the stance phase during which time the limb of interest was undergoing body weight support. Spatiotemporal variables were also extracted; ground contact time was the duration of the stance period; stride time was the time between affected-side foot contacts; stride frequency was the inverse of stride time; stride length was the walking velocity multiplied by stride time. Instantaneous peak isokinetic knee extensor torque at 60° sec^−1^ was obtained from the previously reported dataset (Johansen et al., 2023) to assess associations between peak extensor moments (torques) during walking and peak isokinetic torques. The dynamometer velocity of 60° sec^−1^ was the lowest used in the previous study, and was selected for analysis here as it was deemed to be most relevant to walking (Mentiplay et al., 2018).

### Statistical analysis

Statistical analyses were conducted in MATLAB to compare discrete variables between groups. Normality was assessed through visualisation of Q-Q plots and histograms and based on this assessment, all statistical analyses were non-parametric; Mann–Whitney U-tests assessed differences between independent groups. Separate tests were conducted across the three speed comparisons of interest–Preferred Speed, Resection Group’s Preferred Speed compared to Control Group’s matched speed, and Resection Group’s Fast Speed compared to Control Group’s matched speed. Median and interquartile range (IQR) values were reported per group for each dependent variable. One-tailed tests were conducted for knee flexion, internal knee extensor moment, and negative knee power, where we hypothesised that these values would be lower in the Resection Group than in controls. For all other variables, two-tailed tests were conducted. Sagittal plane kinematics and kinetics at the ankle and hip joint, as well as the contralateral limb, were extracted to further aid the understanding of potential mechanisms. It was deemed more appropriate to conduct statistical analyses of the discrete variables and not the time series data as the differences in magnitude were of most importance to the research questions. Statistical parametric mapping (SPM) analysis could provide valuable insight into the differences throughout stance but requires time-normalisation of the stance phase which was of different durations for each participant. It was not appropriate to conduct SPM analyses in addition to discrete comparisons; thus, time series data were presented for qualitative assessment only. Pearson correlation analyses were conducted to assess correlations between peak extensor torques during walking and peak isokinetic torques. A value of *P* < 0.05 indicated significance for all analyses.

## Results

### Patient characteristics

Data from one participant was excluded due to missing marker data in the static trial. Group mean (standard deviation) characteristics from all other participants are presented in Table 1. Two participants had erroneous marker data at one speed each (one in the Control Group at the fastest speed; one in the Resection Group at the slowest speed) and so were removed from the analysis at those speeds only.

**Table 1:** Participant characteristics. Values are presented as relative numbers and mean (standard deviation).

	Controls	All patients	Distal Femur	Proximal tibia
Female/ male	10/8	10/7	8/1	2/6
Age (years)	31.0 (11.7)	30.6 (12.0)	34.3 (9.7)	26.3 (13.4)
Body weight (kg)	72.9 (10.9)	72.0 (14.2)	72,2 (15,0)	71.8 (14.2)
Height (cm)	174.5 (7.3)	172.6 (9.7)	166.8 (5.4)	179.2 (9.4)
Time since surgery (months)		94.9 (75.8)	106.7 (75.3)	81.7 (79.3)
Operated side left/right		6/11	5/4	5/3
Prosthetic length (cm)		15.1 (4.8)	16.6 (5.9)	13.5 (2.8)
Surgical access medial/ lateral		10/7	3/6	7/1
Chemotherapy yes/no		14/3	7/2	7/1
TESS		81.9 (7.6)	82.4 (8.3)	81.4 (7.2)

**Notes.**

TESS, Toronto Extremity Salvage Score.

#### How do gait variables differ between groups when walking at a preferred speed?

**Figure 1:**
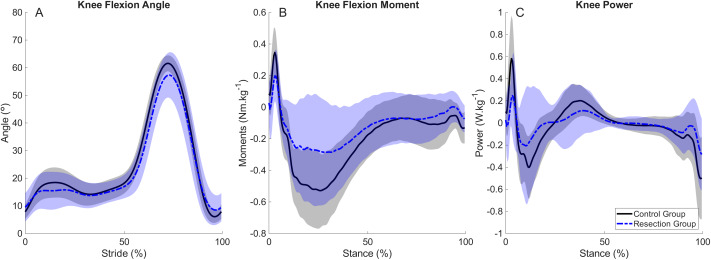
Mean and standard deviation (shading) time series for control and resection groups at each group’s preferred speed, representing (A) knee flexion angle during each stride cycle; (B) Knee flexion moment during stance; (C) knee joint power during stance. Peak knee flexion during stance occurs at approximately 15% of the stride cycle. Peak knee extensor moment and negative power occur at approximately 25% and 10% of stance, respectively.

Spatiotemporal and knee kinematic and kinetic variables are summarised for each group at their preferred walking speeds in Table 2. The Resection Group walked with a preferred walking speed (median (IQR) 0.85 (0.76–0.97) m s^−1^) which was significantly slower than the preferred speed of the Control Group (1.05 (0.96–1.22) m s^−1^). Ground contact time was longer in the Resection Group, (0.74 (0.68–0.80) s) compared with the Control Group (0.68 (0.64–0.72) s), as was stride time (Resection Group: 1.19 (1.12–1.31) s; Control Group: 1.13 (1.0–1.18) s). Stride length was shorter in the Resection Group (1.04 (0.94–1.09) m) than in the Control Group (1.19 (1.11–1.34) m), and stride frequency was lower (Resection Group: 100 (92–108) strides min^−1^; Control Group: 106 (101–110) strides min^−1^). Peak knee flexion angle was not different between groups. Peak knee extensor moments and peak knee joint power were significantly lower in patients than in controls at their preferred walking speed (Fig. 1, Table 2). Time series figures displaying ankle and hip kinematics and kinetics and ground reaction forces for each group at the preferred speed are presented in Figs. S1 and S2, respectively.

**Table 2:** Median and IQR values for spatiotemporal variables and knee kinematics and kinetics for the control group and resection group when walking at their self-selected preferred walking speed.

	**Control group (*n* = 18)**		**Resection group (*n* = 17)**		***p***
	**Median**	**IQR**	**Median**	**IQR**	
**Spatiotemporal variables**					
Walking speed (m s^−1^)	1.05	0.96, 1.22	0.85	0.76, 0.97	**<0.001**
Ground contact time (s)	0.68	0.64, 0.72	0.74	0.68, 0.80	**0.012**
Stride time (s)	1.13	1.09, 1.18	1.19	1.12, 1.31	**0.046**
Stride length (m)	1.19	1.11, 1.34	1.04	0.94, 1.09	**<0.001**
Stride Freq* (Strides min^−1^)	106	101, 110	100	92, 108	**0.046**
**Knee kinematics and kinetics**					
Peak knee flexion angle (^∘^)	19.8	18.1, 21.7	16.1	12.4, 21.6	0.142
Peak knee extensor moments (Nm kg^−1^)	−0.61	−0.45, −0.75	−0.32	−0.48, −0.21	**0.003**
Peak knee joint power (W kg^−1^)	−0.41	−0.30, −0.65	−0.24	−0.30, −0.08	**0.004**

**Notes.**

IQR values are the 25th and 75th percentile values separated by a comma. Bold *P* values indicate a significant difference (*P* < 0.05) between groups.

**Figure 2:**
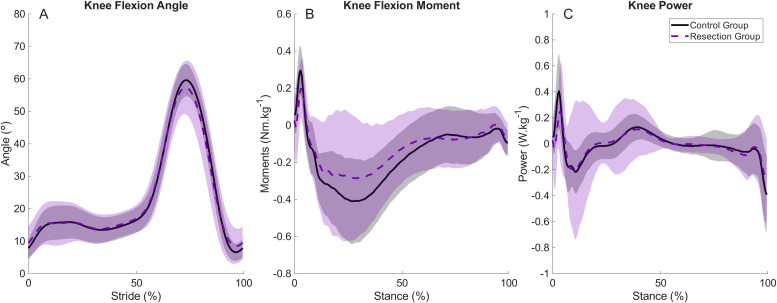
Mean and standard deviation (shading) time series for control and resection groups at the resection group’s preferred speed. Representing (A) knee flexion angle during each stride cycle; (B) Knee flexion moment during stance; (C) knee joint power during stance. Peak knee flexion during stance occurs at approximately 15% of the stride cycle. Peak knee extensor moment and negative power occur at approximately 25% and 10% of stance, respectively.

#### Assessment of walking gait at matched speeds

The Resection Group’s preferred speed (0.85 m s^−1^) was not significantly different from the Control Group’s 80% speed (0.84 m s^−1^). Similarly, there was no significant difference between the Control Group’s preferred speed (1.05 m s^−1^) and the Resection Group’s 120% speed (1.02 m s^−1^), and therefore the groups were compared at these matched speeds (Resection Group Preferred *vs* Control Group 80%, and Resection Group 120% *vs* Control Group Preferred) without exclusion of any participants from these analyses. This allowed for comparison of gait variables between the groups at two matched speeds, referred to as the Resection Group’s Preferred Speed and the Resection Group’s Fast Speed, respectively.

#### How do gait variables differ between groups when walking at the Resection Group’s Preferred Speed?

There were no differences in spatiotemporal variables between groups when walking at the Resection Group’s Preferred Speed (Table 3). Peak knee flexion angle during stance was not different between groups (Fig. 2A, Table 3), whereas peak knee extensor moments were lower in the Resection Group (−0.32 (−0.48 to −0.21) Nm kg^−1^) than the Control Group (−0.45 (−0.58 to −0.38) Nm kg^−1^, Fig. 2B, Table 3), but there was no difference in peak knee joint power between groups (Fig. 2C, Table 3). There were no differences in peak sagittal plane ankle or hip variables (Fig. S3), and no differences in peak anterior-posterior or vertical ground reaction forces (Fig. S4).

**Table 3:** Median and IQR values for spatiotemporal variables and knee kinematics and kinetics for the control group and resection group when walking at the resection group’s preferred speed (0.85 m s^−1^).

	**Control group (*n* = 18)**		**Resection group (*n* = 17)**		***p***
	**Median**	**IQR**	**Median**	**IQR**	
**Spatiotemporal variables**					
Walking speed (m s^−1^)	0.84	0.77, 0.98	0.85	0.76, 0.97	0.960
Ground contact time (s)	0.76	0.73, 0.80	0.74	0.68, 0.80	0.438
Stride time (s)	1.24	1.18, 1.30	1.19	1.12, 1.31	0.805
Stride length (m)	1.04	0.96, 1.16	1.04	0.94, 1.09	0.656
Stride Freq* (Strides min^−1^)	97	93, 101	100	92,108	0.805
**Knee kinematics and kinetics**					
Peak knee flexion angle (^∘^)	17.2	14.8, 19.6	15.3	11.8, 20.8	0.390
Peak knee extensor moments (Nm kg^−1^)	−0.45	−0.58, −0.38	−0.32	−0.48, −0.21	**0.045**
Peak knee joint power (W kg^−1^)	−0.25	−0.47, −0.19	−0.24	−0.30, −0.08	0.209

**Notes.**

IQR values are the 25th and 75th percentile values separated by a comma. Bold *P* values indicate a significant difference (*P* < 0.05) between groups.

#### How do gait variables differ between groups when walking at the Resection Group’s Fast Speed?

**Figure 3:**
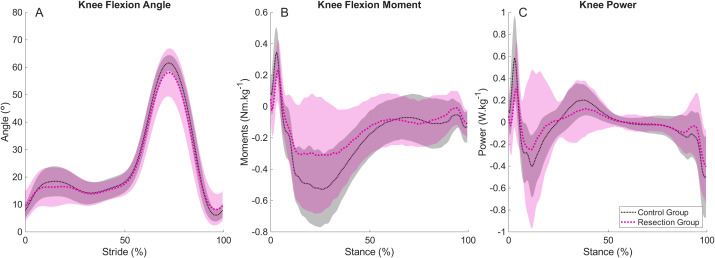
Mean and standard deviation (shading) time series for control and resection groups at the resection group’s fast speed, representing (A) knee flexion angle during each stride cycle; (B) knee flexion moment during stance; (C) knee joint power during stance. Peak knee flexion during stance occurs at approximately 15% of the stride cycle. Peak knee extensor moment and negative power occur at approximately 25% and 10% of stance, respectively.

There were no differences in spatiotemporal variables between groups (Table 4) and no differences in peak knee flexion angle during stance between groups (Fig. 3A, Table 4) when walking at the Resection Group’s Fast Speed. Peak knee joint moments were lower in the Resection Group (−0.36 (−0.54 to −0.25) Nm kg^−1^) than Control Group (−0.61 (−0.75 to −0.45) Nm kg^−1^) and peak knee joint power was also lower in the Resection Group (−0.23 (−0.30 to −0.07) W kg^−1^) than in the Control Group (−0.41 (−0.65 to −0.31) W kg^−1^) (Fig. 3B, Fig. 3C, Table 4). There were no differences in peak sagittal plane ankle or hip angles or moments (Fig. S5). Initial peak vertical force was lower in the Resection Group (median (IQR) 0.98 (0.96, 1.04) BW) than the Control Group (1.04 (1.01, 1.07) BW, *p* = 0.028, Fig. S6A). Peak braking forces were also lower in the Resection Group (−0.11 (−0.14, −0.09) BW) than the Control Group (−0.14 (−0.16, −0.12) BW, *p* = 0.018, Fig. S6B), but there were no differences in propulsive forces between groups (*p* = 0.204).

**Table 4:** Median and IQR values for spatiotemporal variables and knee kinematics and kinetics for the control group and resection group when walking at the resection group’s fast speed (1.03 m s^−1^).

	**Control group (*n* = 18)**		**Resection group (*n* = 17)**		***p***
	**Median**	**IQR**	**Median**	**IQR**	
**Spatiotemporal variables**					
Walking speed (m s^−1^)	1.05	0.96–1.22	1.02	0.92–1.16	0.467
Ground contact time (s)	0.68	0.64, 0.72	0.67	0.62, 0.73	0.754
Stride time (s)	1.13	1.09, 1.18	1.11	1.04, 1.19	0.754
Stride length (m)	1.19	1.11, 1.34	1.12	1.08, 1.22	0.171
Stride Freq* (Strides min^−1^)	106	101, 110	108	101, 115	0.704
**Knee kinematics and kinetics**					
Peak knee flexion angle (^∘^)	19.4	17.0, 21.7	16.0	12.1, 22.7	0.142
Peak knee extensor moments (Nm kg^−1^)	−0.61	−0.75, −0.45	−0.36	−0.54, −0.25	**0.014**
Peak knee joint power (W kg^−1^)	−0.41	−0.65, −0.31	−0.23	−0.30, −0.07	**0.007**

**Notes.**

IQR values are the 25th and 75th percentile values separated by a comma. Bold *P* values indicate a significant difference (*P* < 0.05) between groups.

### Associations between knee extensor moments during walking and dynamometer-obtained knee extension torque

There was a significant, moderate inverse association between peak isokinetic knee extension torque at 60° sec^−1^ and the magnitude of peak knee extension torque during walking at the Resection Group’s Preferred Speed (*r* = −0.511, *p* = 0.036), indicating that patients who generated greater isokinetic torques exhibited lower magnitudes of internal knee extensor moments during walking. There was no association between these variables in the Control Group (*r* = 0.157, *p* = 0.533) at the Resection Group’s Preferred Speed.

## Discussion

**Figure 4:**
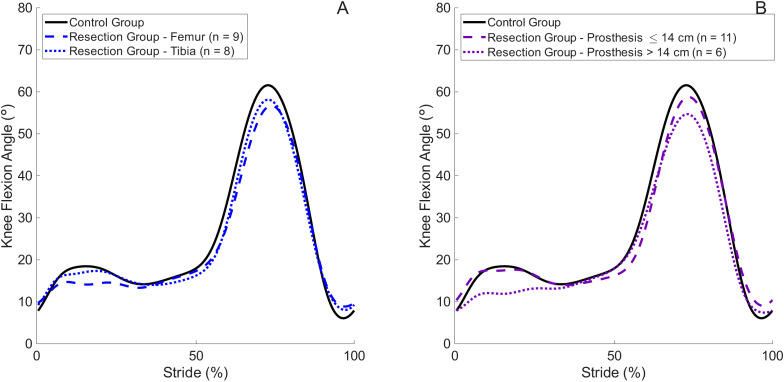
Group mean knee flexion angle during the stride cycle for the Control Group and Resection Group walking at each individual’s preferred walking speed, where the Resection Group has been subdivided according to (A) tumour location; (B) length of prosthesis. n value represents the number of patient participants in each subgroup. Peak knee flexion during stance occurs at approximately 15% of the stride cycle.

Individuals who have undergone prosthetic reconstruction after tumour resection had a slower preferred walking speed than healthy controls, which was achieved through a combination of a shorter stride length and slower stride frequency, as previously observed (Filis et al., 2022) and in support of our hypothesis. The slower walking speed and accompanying shorter stride length in patients in the present study likely explain the lower knee joint kinetics observed in the Resection Group than controls when walking at their own preferred speed, in support of previous findings (Chen, Kuo & Andriacchi, 1997; Holden, Chou & Stanhope, 1997). However, there was no significant difference in knee flexion angle during stance between these groups, in contrast to previous findings (Carty et al., 2009). It has previously been reported that tumour resection patients display either an excessively extended knee angle during early stance or a more flexed pattern compared with healthy controls (Carty et al., 2009; Rompen et al., 2002). A possible explanation for this heterogeneity among tumour resection patients was previously proposed to relate to the inclusion of both patients with a femoral resection and a tibial resection (Carty et al., 2009). In the present data set it appeared that patients demonstrated a different knee flexion pattern during stance (first 20% of stride) than controls, visible in Fig. 1A. To explore this further and to assess whether this may have been influenced by the nature of the resection, the Resection Group was divided into femoral resection and tibial resection subgroups (Fig. 4A). We separately divided the dataset into subgroups based on the length of the prosthesis: ≤14 cm (*n* = 11) and >14 cm (*n* = 6, Fig. 4B). Prosthesis length was used as an available surrogate measure of the extent of the surgical procedure, and the cutoff of 14 cm was chosen to create distinct groups in terms of average prosthesis length. From the present dataset, patients with a prosthesis length ≤ 14 cm display qualitatively more similar knee flexion patterns to controls at the preferred walking speed, whereas those with a longer prosthesis display the same limited knee flexion in early stance as reported by Carty et al. (2009). This trend was more pronounced than a division by tumour location. Importantly, the present study was not designed or powered to identify differences between subgroups, but further analysis of this and the mechanisms that may explain it are warranted.

Given that walking speed and its relationship to stride length are known to influence knee joint mechanics, it was important to quantify and compare knee joint mechanics between groups when walking at matched speeds. In the present study, it was possible to compare walking gait at two matched walking speeds: one that represented the Resection Group’s Preferred Speed and another representing the Resection Group’s Fast Speed. Despite no differences in spatiotemporal characteristics between the two groups when walking at matched speeds, the Resection Group demonstrated ∼29% and ∼41% lower knee extensor moments than the Control Group at the Preferred and Fast Speeds, respectively. The Resection Group also demonstrated lower knee power at the Fast Speed, but no differences in knee joint kinematics at either speed, in partial support of the hypotheses. These findings also support the hypothesis that the differences between groups would be exacerbated at faster walking speeds. The lower magnitude knee extensor moments observed suggest that the Resection Group utilised a different strategy from the Control Group to achieve the equivalent walking speed. This could have been achieved by a greater mechanical contribution from the contralateral, unaffected limb, or from the other lower limb joints. The results demonstrate that there were no differences between groups in terms of sagittal plane ankle or hip mechanics on the affected side, which indicates that patients were not relying on a relatively greater mechanical contribution from the ankle or hip on this side. Supplementary analyses to assess whether joint kinetics were greater on the Resection Group’s unaffected side than the affected side were conducted (non-parametric one-tailed Wilcoxon Signed Rank tests) and revealed no differences between sides at the Preferred Speed. However, there were ∼9% greater ankle plantarflexor moments in the unaffected side than the affected side at the Fast Speed (*p* = 0.034), and ∼24% greater (non-significant, *p* = 0.097) knee extensor moments, in line with observations by Okita et al. (2014), but no other differences. It should be noted that there was much greater variability in kinematics and kinetics of the Resection Group than the Control Group, particularly evident at the time of peak knee extensor moments in early stance. It is likely that patients relied on a combination of increased mechanical work contributions from the ankle and hip joint of the affected limb and the ankle, knee, and hip joints of the contralateral limb, but that a variety of inter-individual strategies were adopted such that clear systematic differences were not observed in the data. It must also be noted that although the average walking speeds compared here were not different, these walking speeds were not pre-determined, and thus the data were obtained from a range of walking speeds.

The lower magnitude knee joint kinetics observed in the Resection Group during walking may be a mechanism to protect the knee joint or may be due to an inability to generate the higher forces demonstrated by the Control Group, due potentially to insufficient muscular force-generating capabilities or altered neuromuscular control. Assessing associations between isokinetic knee extensor torques and peak knee extensor moments during walking provides some insight into this. Mean peak isokinetic knee extension torques at 60° sec^−1^ were 2.45 Nm kg^−1^ in the Control Group and 0.45 Nm kg^−1^ in the Resection Group from the same dataset (Johansen et al., 2023) (normalised to body mass for comparison with values during walking). During walking, the median peak knee extension moments were 0.45 Nm kg^−1^ in the Control Group and 0.32 Nm kg^−1^ in the Resection Group at the Resection Group’s Preferred Speed, and 0.61 and 0.36 Nm kg^−1^, respectively, at the Fast Speed. However, peak knee extensor torque magnitudes are well-understood to be lower when the knee is closer to full extension than when flexed (*e.g.*, Haffajee, Moritz & Svantesson, 1972), and therefore during walking stance—when the knee remains relatively extended—it would be assumed that the Resection Group would be able to generate knee extension moments of less than 0.45 Nm kg^−1^ on average. This shows that walking is mechanically much more demanding for patients relative to their maximal capacity than it is for controls. Hence, many patients may not have the knee extension strength required to generate the greater knee joint kinetics that faster walking requires; thus, other strategies must be adopted.

**Figure 5:**
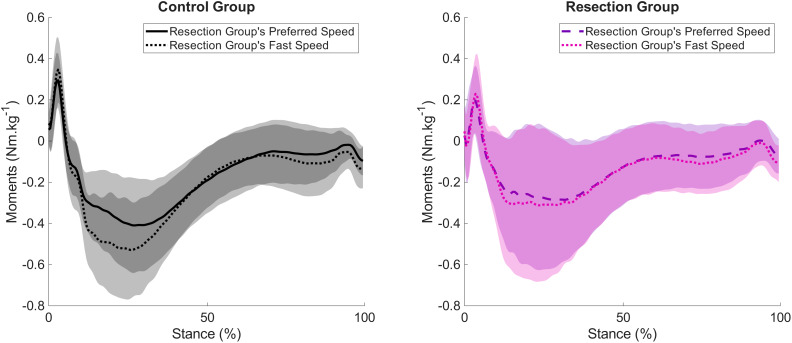
Mean and standard deviation (shading) knee flexion moment during stance for the control group (left) and resection group (Right) at the speeds equivalent to the Resection Group’s Preferred and Fast Speed.

The apparent inability to generate greater knee joint kinetics to achieve faster walking can be observed by considering the within-group differences in knee joint moments demonstrated as speed increased from Patient’s Preferred to Patient’s Fast Speed (Fig. 5). When increasing speed by 20%, the Control Group demonstrated an approximate increase in peak knee extension moment of ∼28%, whereas the Resection Group demonstrated an average increase of ∼8%. As discussed, to generate the faster walking speeds achieved, alternative strategies must have been adopted, including greater reliance on the unaffected side. The participants in the present study were pain-free during walking, and as such, it seems that a lack of extensor strength predominantly explains the lower magnitude of knee joint kinetics observed, rather than this being a protective mechanism to minimise pain or reduce loading on a prosthetic knee.

It is also noteworthy that peak braking forces were 27% lower in the Resection Group than the Control Group when walking at the Fast Speed, despite the speeds being matched. The time of peak braking force approximately coincides with the time of peak knee extensor moment and negative power, and thus likely relates to the mechanisms for lower knee kinetics observed. It has previously been shown that peak braking forces can be reduced during running whilst maintaining the same speed by reducing stride length and increasing stride frequency (Napier et al., 2019). In the present study, there was no significant difference in these characteristics, but a 6% non-significantly lower stride length in the Resection Group was observed. In line with our above suggestion that a variety of strategies were adopted, we speculate that one such strategy adopted by the Resection Group was to take shorter strides at the Fast Speed in order to meet the demands of the faster walking speed without considerably increasing eccentric loading of the knee in early stance.

The moderate inverse association between isokinetic knee extensor torque and peak knee extensor moments during walking in the Resection Group was in contrast to the hypothesis. The heterogeneity of the group makes interpretation of this difficult: three patients generated torques that were less than 6% of the Control Group’s average value, *i.e.,* almost negligible in magnitude. When combining the data from the Resection Group and Control Group, there was no association between these variables (*r* = 0.061, *p* = 0.728).

### Limitations

This study provides novel insight into the potential mechanisms by which walking gait may be impaired in Resection Patients by comparing with healthy controls at speeds that were relative to each individual’s preferred walking speed. This allowed inclusion of a heterogeneous participant sample, but means that the speeds were not identical between participants or groups. Furthermore, when comparing gait at matched walking speeds, this represents a less challenging task for control participants than patients who had undergone resection. For participants in both groups, walking on a treadmill constrains the participants and introduces an element of unfamiliarity that means it is likely not representative of the true walking gait that participants adopt on a daily basis.

The heterogeneity of the Resection Group, in particular, means that certain mechanisms that occur may not have been detected. This is especially relevant in the case of potentially dichotomous knee flexion strategies observed within the Resection Group that effectively cancel each other out when averaging for statistical analysis. Furthermore, whilst the study was powered to test the main hypotheses, it was limited in terms of further analysis of sub-groups, and this limits the interpretation of the correlation analysis in particular.

Limitations are also inherent in the assessment of lower limb kinematics and kinetics using a generic model, developed from existing data that does not account for differences that may occur as a result of limb resection. Joint moment arms and joint centre locations derived from marker locations are likely different following limb resection than in control participants. The influence of such errors on estimating joint kinetics in the patient population is unknown.

### Implications

The evidence from this study, combined with previous findings from the same participants (Johansen et al., 2023), suggests that individuals who have undergone prosthetic reconstruction after tumour resection demonstrate lower magnitude sagittal plane knee joint kinetics than controls, possibly as a result of impaired knee extensor capability, driven by reduced quadriceps muscular function following resection. This hypothesis warrants investigation, for example through assessment of the influence of knee extensor assistive exoskeletons on walking gait, and randomised controlled trials that assess the efficacy of targeted knee extensor rehabilitation interventions on short- and long-term outcomes.

## Conclusions

Patients who have undergone prosthetic reconstruction after tumour resection walk at a slower preferred speed than matched healthy controls, which influences knee joint kinetics. Furthermore, even when walking at matched speeds, peak sagittal plane knee joint kinetics are lower in patients than in controls, and this is exacerbated at faster walking speeds. This is likely due to the patients’ inability to generate the required knee extensor forces. It appears that a sub-group of patients categorised according to prosthesis length exists, who demonstrate an extended knee during early stance, and further investigation of their mechanics is warranted.

## Supplemental Information

10.7717/peerj.21201/supp-1 Supplemental Information 1 Supplementary Figures

10.7717/peerj.21201/supp-2 Supplemental Information 2 Walking Speed Control Group Data columns represent each walking speed (Speed 1 = Slower; Speed 2 = Preferred, Speed 3 = Faster) with mean values for each participant presented

10.7717/peerj.21201/supp-3 Supplemental Information 3 Walking Speed Patients Data columns represent each walking speed (Speed 1 = Slower; Speed 2 = Preferred, Speed 3 = Faster) with mean values for each participant presented

10.7717/peerj.21201/supp-4 Supplemental Information 4 Stance Time Control Group Data columns represent each walking speed (Speed 1 = Slower; Speed 2 = Preferred, Speed 3 = Faster) with mean values for each participant presented

10.7717/peerj.21201/supp-5 Supplemental Information 5 Stance Time Patients Data columns represent each walking speed (Speed 1 = Slower; Speed 2 = Preferred, Speed 3 = Faster) with mean values for each participant presented

10.7717/peerj.21201/supp-6 Supplemental Information 6 Stride Time Control Group Data columns represent each walking speed (Speed 1 = Slower; Speed 2 = Preferred, Speed 3 = Faster) with mean values for each participant presented

10.7717/peerj.21201/supp-7 Supplemental Information 7 Stride Time Patients Data columns represent each walking speed (Speed 1 = Slower; Speed 2 = Preferred, Speed 3 = Faster) with mean values for each participant presented

10.7717/peerj.21201/supp-8 Supplemental Information 8 Stride Length Control Group Data columns represent each walking speed (Speed 1 = Slower; Speed 2 = Preferred, Speed 3 = Faster) with mean values for each participant presented

10.7717/peerj.21201/supp-9 Supplemental Information 9 Stride Length Patients Data columns represent each walking speed (Speed 1 = Slower; Speed 2 = Preferred, Speed 3 = Faster) with mean values for each participant presented

10.7717/peerj.21201/supp-10 Supplemental Information 10 Stride Frequency Control Group Data columns represent each walking speed (Speed 1 = Slower; Speed 2 = Preferred, Speed 3 = Faster) with mean values for each participant presented

10.7717/peerj.21201/supp-11 Supplemental Information 11 Stride Frequency Patients Data columns represent each walking speed (Speed 1 = Slower; Speed 2 = Preferred, Speed 3 = Faster) with mean values for each participant presented

10.7717/peerj.21201/supp-12 Supplemental Information 12 Peak Knee Flexion Control Group Data columns represent each walking speed (Speed 1 = Slower; Speed 2 = Preferred, Speed 3 = Faster) with mean values for each participant presented

10.7717/peerj.21201/supp-13 Supplemental Information 13 Peak Knee Flexion Patients Data columns represent each walking speed (Speed 1 = Slower; Speed 2 = Preferred, Speed 3 = Faster) with mean values for each participant presented

10.7717/peerj.21201/supp-14 Supplemental Information 14 Peak Knee Power Control Group Data columns represent each walking speed (Speed 1 = Slower; Speed 2 = Preferred, Speed 3 = Faster) with mean values for each participant presented

10.7717/peerj.21201/supp-15 Supplemental Information 15 Peak Knee Moments Patients Data columns represent each walking speed (Speed 1 = Slower; Speed 2 = Preferred, Speed 3 = Faster) with mean values for each participant presented

10.7717/peerj.21201/supp-16 Supplemental Information 16 Peak Knee Moment Control Group Data columns represent each walking speed (Speed 1 = Slower; Speed 2 = Preferred, Speed 3 =Faster) with mean values for each participant presented

10.7717/peerj.21201/supp-17 Supplemental Information 17 Peak Knee Power Patients Data columns represent each walking speed (Speed 1 = Slower; Speed 2 = Preferred, Speed 3 = Faster) with mean values for each participant presented

## Additional Information and Declarations

### Data Availability

The following information was supplied regarding data availability: The time series kinematic and kinetic raw data is available in the Supplemental Files.
