# Supplementary material for: Lower peak knee joint kinetics during walking in patients with knee reconstruction for bone sarcoma compared to healthy controls
Source: PeerJ. 2026 May 6;14:e21201. doi: 10.7717/peerj.21201 (PMC13156953; doi:10.7717/peerj.21201)
Supplement: Supplemental Information 1 [file peerj-14-21201-s001.docx]

***Aim 1: How do gait variables differ between groups when walking at a preferred speed?***


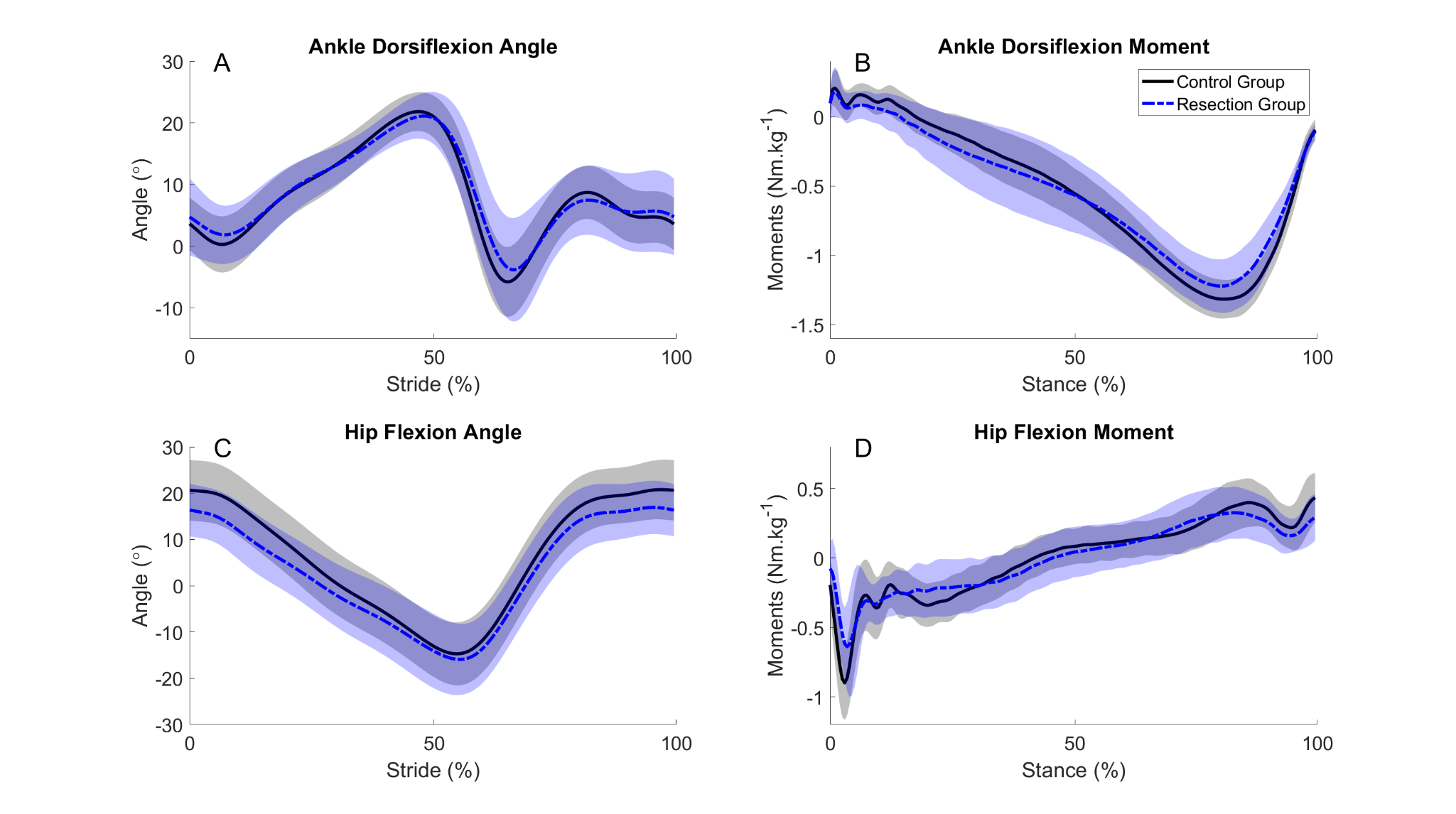


Figure 1: Mean and standard deviation (shading) time series for Control and Resection Groups walking at their preferred speeds. A) Ankle dorsiflexion angle during each stride; B) Ankle dorsiflexion during stance; C) Hip flexion angle during each stride; D) Hip flexion moment during stance. Positive values represent the direction corresponding to the figure title (e.g. positive values in A represent dorsiflexion; negative values represent plantarflexion). Joint moments are represented as internal moments.


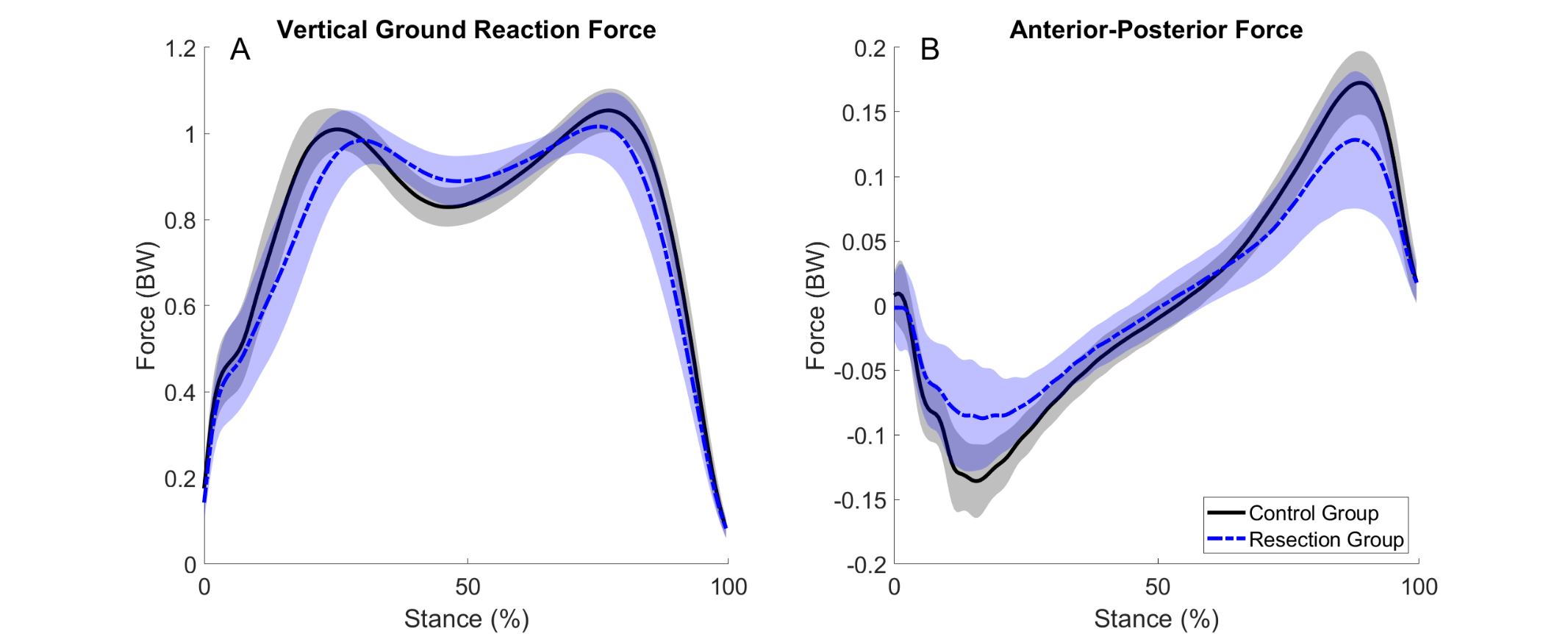


Figure 2: Mean and standard deviation (shading) time series for Control and Resection Groups walking at their preferred speeds. A) Vertical ground reaction forces during stance; B) Anterior-posterior ground reaction forces during stance.

***Aim 2: How do gait variables differ between groups when walking at the Resection Group’s Preferred Speed?***


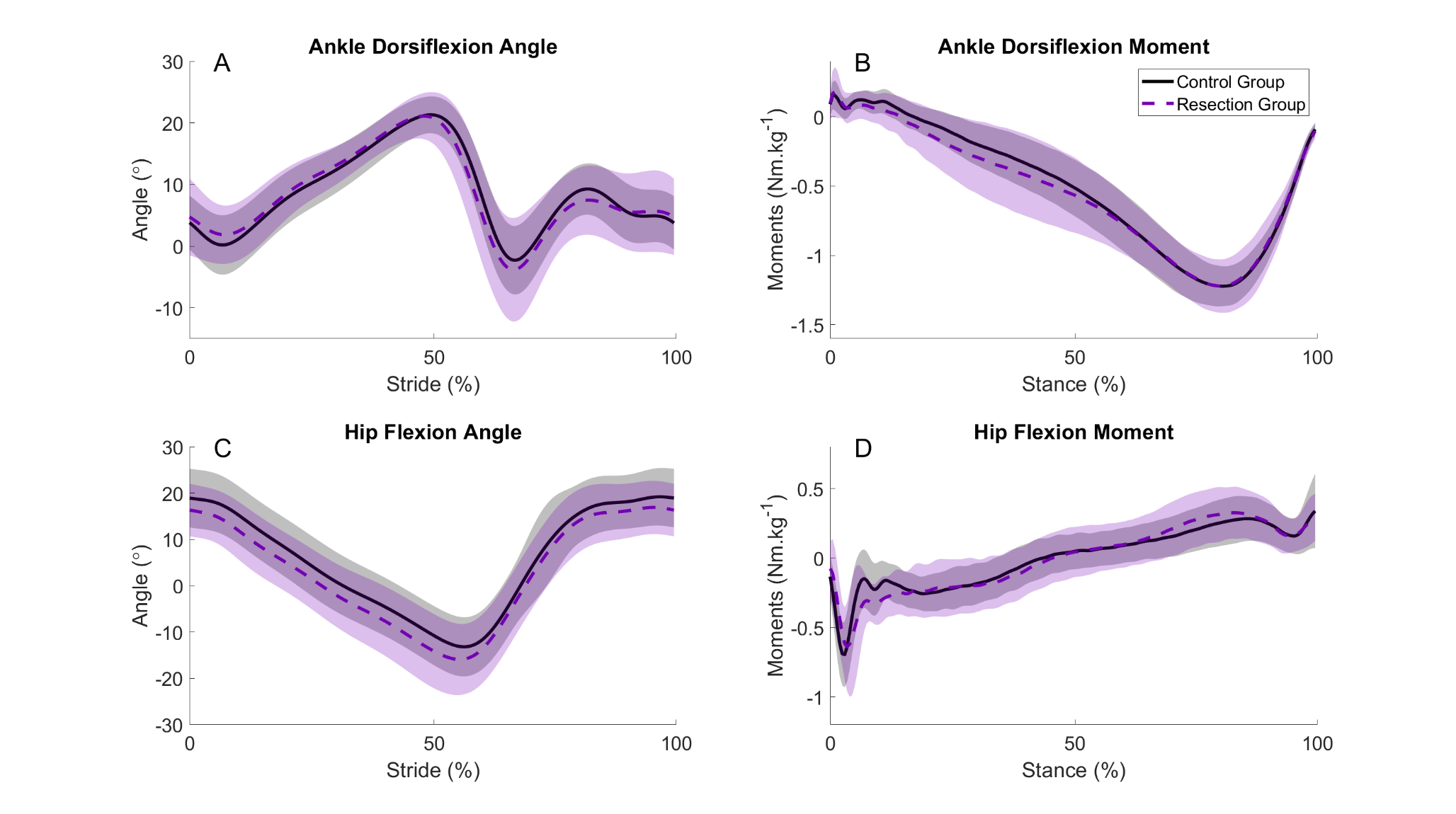


Figure 3: Mean and standard deviation (shading) time series for Control and Resection Groups during walking at the Resection Group’s Preferred Speed. A) Ankle dorsiflexion angle during each stride; B) Ankle dorsiflexion during stance; C) Hip flexion angle during each stride; D) Hip flexion moment during stance. Positive values represent the direction corresponding to the figure title (e.g. positive values in A represent dorsiflexion; negative values represent plantarflexion). Joint moments are represented as internal moments.


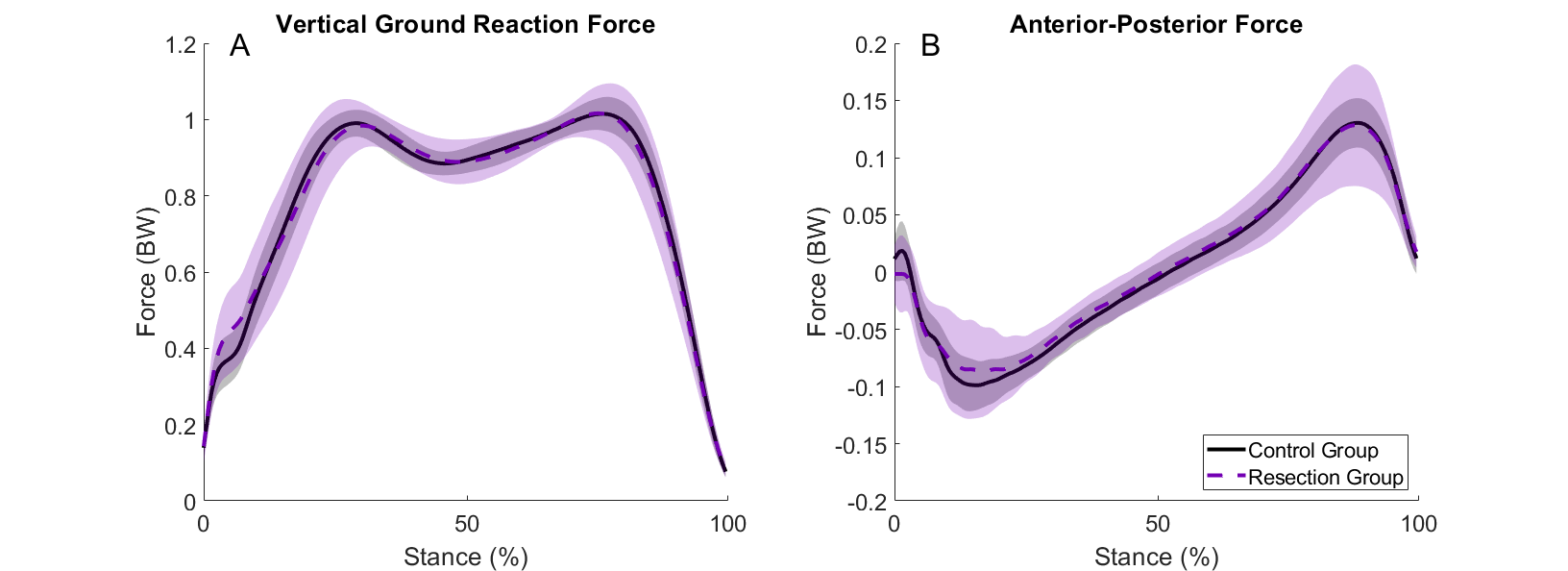


Figure 4: Mean and standard deviation (shading) time series for Control and Resection Groups walking at Resection Group’s Preferred Speed. A) Vertical ground reaction forces during stance; B) Anterior-posterior ground reaction forces during stance.

***Aim 3: How do gait variables differ between groups when walking at the Resection Group’s Fast Speed?***


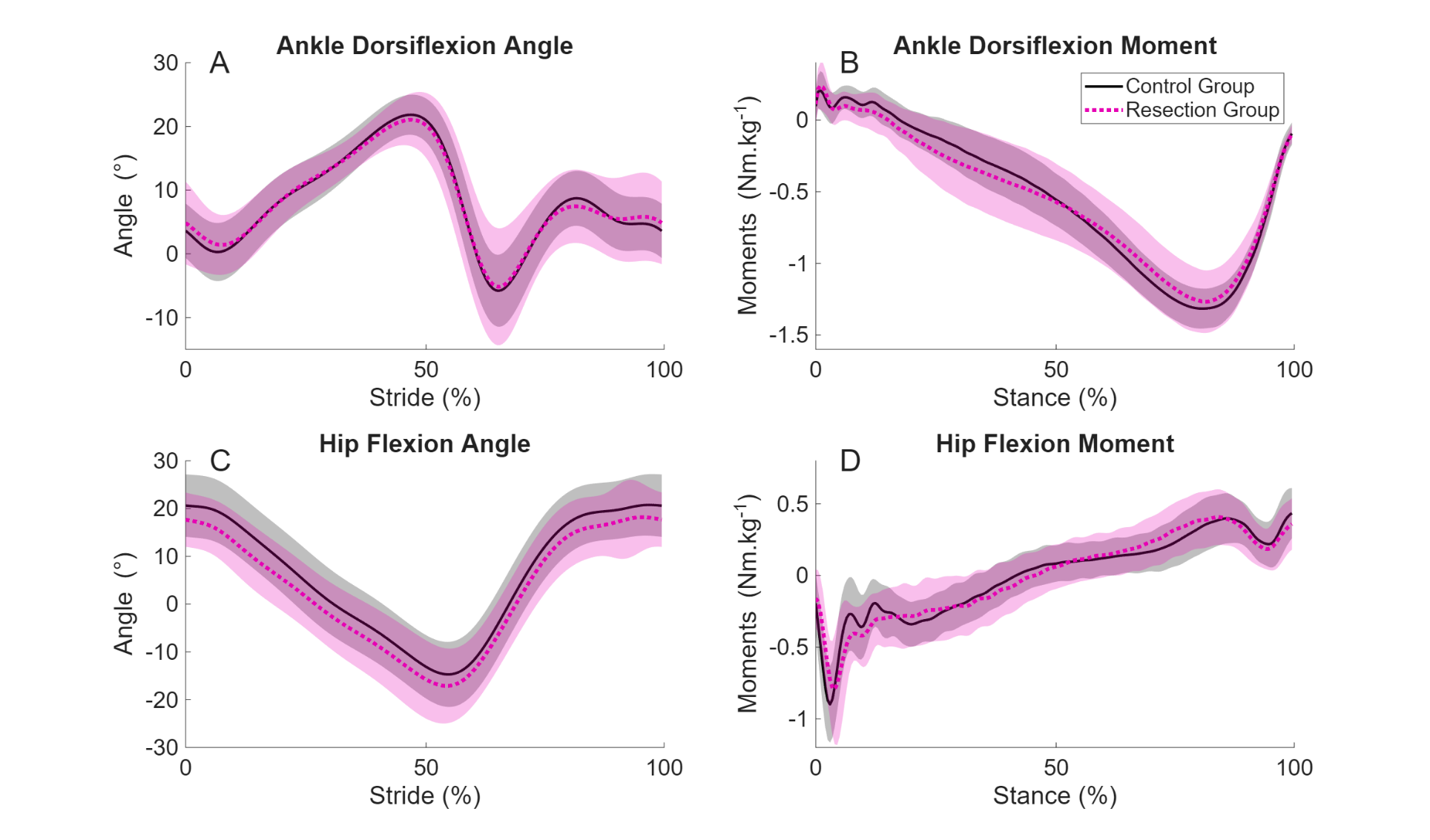


Figure 5: Mean and standard deviation (shading) time series for Control and Resection Groups during walking at the Resection Group’s Fast Speed. A) Ankle dorsiflexion angle during each stride; B) Ankle dorsiflexion during stance; C) Hip flexion angle during each stride; D) Hip flexion moment during stance. Positive values represent the direction corresponding to the figure title (e.g. positive values in A represent dorsiflexion; negative values represent plantarflexion). Joint moments are represented as internal moments.


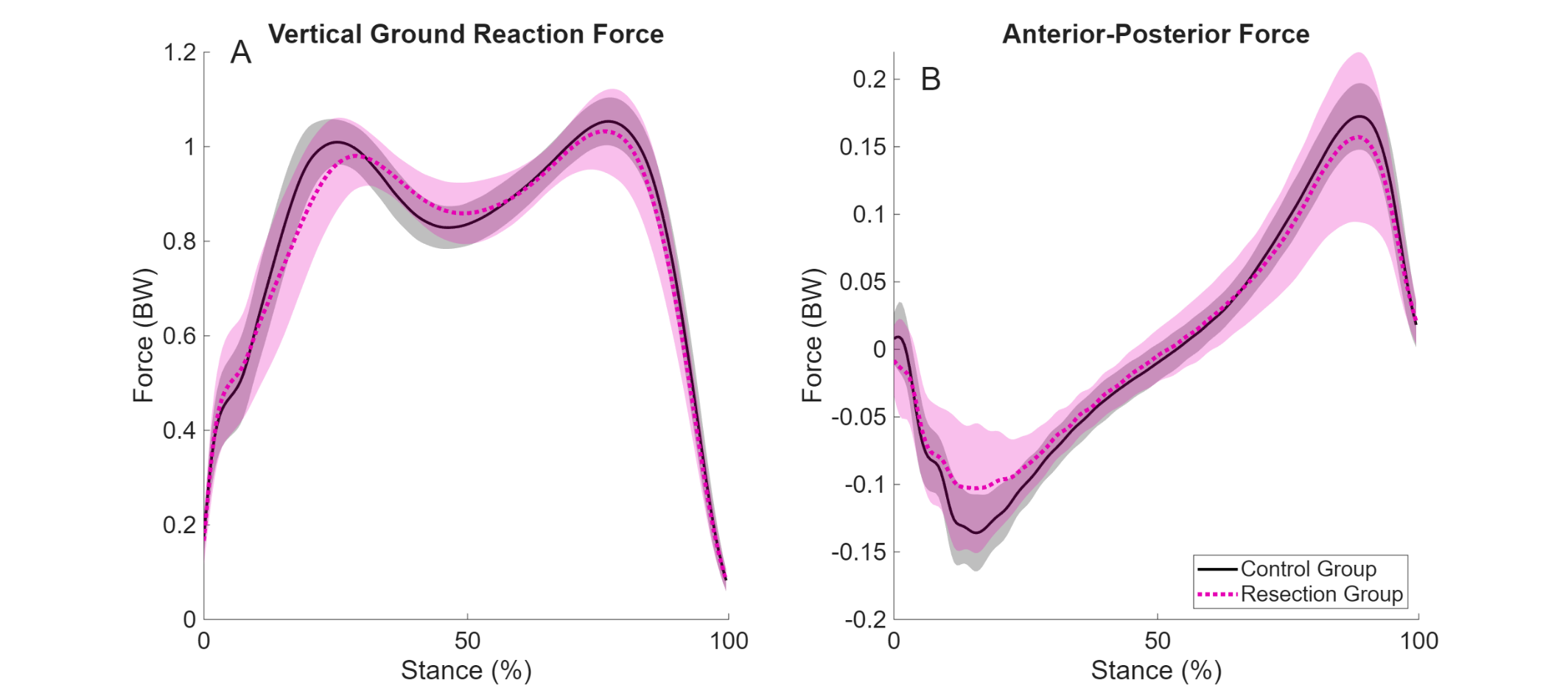


Figure 6: Mean and standard deviation (shading) time series for Control and Resection Groups walking at Resection Group’s Fast Speed. A) Vertical ground reaction forces during stance; B) Anterior-posterior ground reaction forces during stance.
